# Cellulose Acetate-Based Membranes Recovered from Black-and-White Cinematographic Films for the Simultaneous Removal of Nitrate and Phosphate Anions from Water by Nanofiltration

**DOI:** 10.3390/toxics14070640

**Published:** 2026-07-22

**Authors:** Aurelia Cristina Nechifor, Paul Constantin Albu, Alexandra Raluca Grosu, Geani-Teodor Man, Vlad-Alexandru Grosu

**Affiliations:** 1 Analytical Chemistry and Environmental Engineering Department, Faculty of Chemical Engineering and Biotechnologies, National University of Science and Technology POLITEHNICA Bucharest, 1–7 Gheorghe Polizu, 011061 Bucharest, Romania; aureliacristinanechifor@gmail.com (A.C.N.); andra.grosu@upb.ro (A.R.G.); 2 Radioisotopes and Radiation Metrology Department (DRMR), IFIN Horia Hulubei, 023465 Măgurele, Romania; 3 National Research and Development Institute for Cryogenics and Isotopic Technologies–ICSI, 240050 Râmnicu-Vâlcea, Romania; geani.man@icsi.ro; 4 Department of Electronic Technology and Reliability, Faculty of Electronics, Telecommunications and Information Technology, National University of Science and Technology POLITEHNICA Bucharest, 1–3 Iuliu Maniu Blvd., 061071 Bucharest, Romania

**Keywords:** cellulose acetate, silver nanoparticles, composite membranes, nitrate, phosphate, nanofiltration

## Abstract

Among the micropollutants of medium-depth waters in isolated inhabited areas, the inorganic ones deserve special attention: nitrate anion (NO_3_^−^) and phosphate anions (HxPO_4_^−(3−x)^). The individual removal of these anions from water is widely studied, with different methods being found: chemical, ion exchange, or biological. This paper presents a membrane method for the simultaneous removal of nitrate anion and phosphate anions from dilute synthetic aqueous solutions. The developed method is nanofiltration using composite membranes made of cellulose acetate (CA) and silver nanoparticles (Agnp). The composite membranes were made by phase inversion of the dimethylformamide (DMF) solution containing the two components (CA–Agnp) on a polypropylene (PP) capillary fiber using deionized water as a coagulant. The DMF solution of CA containing Agnp was obtained by dissolving black-and-white cinematographic films (exposed and unexposed to light). CA–Agnp–PP composite membranes were tested for the simultaneous removal of nitrate anion and phosphate anions from aqueous solution by nanofiltration at pressures ranging from 5 to 25 bars. A removal of over 98% of phosphate anions and more than 95% of nitrate anions was achieved. Fluxes of 10 L·m^−2^·h^−1^ were obtained for the working pressure of 15 atm, depending on the *p*H, flow rate, and concentration of the feed water. The variable parameters considered were also the concentrations of CA and Agnp.

## 1. Introduction

Membranes and membrane processes involving cellulose derivatives as membrane materials are today a common presence in technologies for obtaining drinking water and treating aqueous effluents [1,2,3].

**Figure 1:**
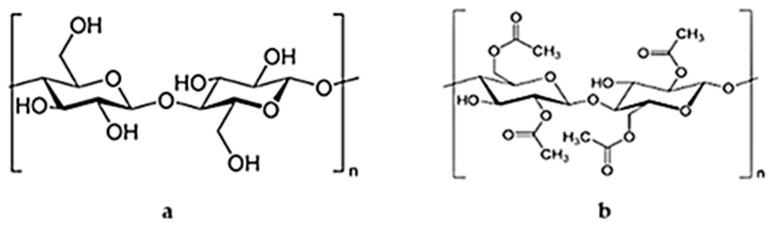
(**a**) Cellulose and (**b**) cellulose acetate.

Cellulose (C) (Figure 1a) is a membrane material with excellent properties [4], but one of its derivatives, cellulose acetate (Figure 1b), still finds wide use today [5,6,7,8].

The success of these raw materials in various technological achievements has led to an unprecedented development of research to obtain membranes with high selectivity, increasingly higher productivity, and long life [9,10].

In recent years, cellulose acetate has once again become the main actor in research to obtain membranes with industrial but also domestic or small-scale applications [11,12,13].

The disadvantages of cellulose acetate, compared with synthetic polymers, have become advantages in the era of clean and cleaning technologies: (i) the selectivity of the artificial polymer and the productivity of the membranes have been largely improved by the contribution of nanomaterials, such as allotropic forms of carbon, metallic, oxide, or composite nanoparticles [14,15,16,17,18]; (ii) lower resistance to high *p*H allows recycling of used membranes by dissolution in strongly alkaline solutions, and filtration is followed by precipitation in water or aqueous solutions; and (iii) biodegradability gives increased attractiveness to cellulose acetate in the case of waste, which thus no longer represents an environmental problem.

The use of silver nanoparticles in cellulose acetate-based membranes is widely studied because it improves catalytic, biocidal, and anti-fouling characteristics [13,15]. However, it should be noted that the presence of silver nanoparticles in membranes can lead to leaching and contamination of the permeate with silver ions, which produces toxicity specific to heavy metals [13,15,19].

An aspect that initially did not constitute a problem, the accessibility and low price of cellulose acetate, has become, in recent years, an issue discussed by ecologists because the enormous quantities of cellulose acetate used worldwide have created the justified feeling that forests are disappearing also due to the production of membranes based on cellulose derivatives.

As expected, the researchers’ solutions came quickly, and recently membranes obtained from newspaper waste [20], photographic and cinematographic films [21], or cigarette filters [22] have been promoted, and ideas for recycling cellulose acetate seem to be in continuous progress [23,24].

Obtaining drinking water in isolated inhabited areas involves the removal of inorganic micropollutants from shallow groundwater: nitrate anion or/and phosphate anions.

While the phosphate anion is most likely to be found in the waters from these areas, the nitrate anion is also undesirable, which often occurs either from agricultural fertilizers or from acid rains [25,26].

The EU regulations are restrictive for nitrate anions because they are aggressive, especially for young children.

The European regulations regarding the phosphate ion focus on reducing water eutrophication and come from several regulatory acts, not a single directive [25,26].

For both the nitrate and phosphate anions, a series of chemical or biological removal methods have been developed [27,28,29]. At the same time, membrane removal methods from water have been studied for these anions [30,31,32,33].

It should be emphasized that studies on many membrane methods have been developed for aqueous solutions containing either the nitrate anion or the phosphate anion [34,35,36].

For the nitrate anion, nanofiltration processes through various types of membranes are well developed, some reaching the commercial stage [37,38,39,40].

This paper presents a method for the simultaneous removal of the proposed ions (nitrate and phosphate) using membranes. The developed method uses nanofiltration through cellulose acetate (CA)–silver nanoparticle (Agnp) composite membranes, obtained from cinematographic films.

The composite membrane (CA–Agnp–PP) was obtained by phase inversion by contacting a dispersion in DMF of the cellulose acetate (CA)–silver nanoparticle (Agnp) assembly on a tubular propylene (PP) membrane.

## 2. Materials, Reagents and Methods

### 2.1. Materials and Reagents

The reagents used were the following: NaNO_3_, (85.00 g/mol) and AgNO_3_ (169.87 g/mol) (Merck KGaA, Darmstadt, Germany), anhydrous disodium phosphate (141.96 g/mol) (99%, Sigma Aldrich, Burlington, MA, USA), dimethylformamide (DMF) (73.09 g/mol) (99%, Honeywell, Charlotte, NC, USA), nitric acid (63.01 g/Mol) (70%) and hydrochloric acid (36.46 g/mol) (37%, Sigma Aldrich, Burlington, MA, USA), and sodium hydroxide (40.00 g/mol) (Merck, Rahway, NJ, USA).

The cellulose acetate used was waste from the film industry (containing silver nanoparticles) [21].

The polypropylene hollow fiber membrane support (PP) was provided by GOST (GOST Ltd., Perugia, Italy), and its characteristics and performance were previously presented: porosity 40–50%, pore dimension 0.02–0.2 µm, fascicle dimensions of 25 mm × 75 mm, and a filtration surface (for ten fascicles) of 1.0 m^2^ [21,41].

Rapid analytical tests performed using LCK349 Phosphorus total/Phosphate ortho and LCK339 Nitrate (Hach Lange GmbH, Düsseldorf, Germany) are specific to the analysis of test components in synthetic solutions prepared in the laboratory (phosphates, nitrates).

The purified water, characterized by 18.2 µS/cm conductivity, was obtained using a RO Millipore system (MilliQ^R^ Direct 8 RO Water Purification System, Merck, Darmstadt, Germany) [21].

### 2.2. Methods and Procedures

#### 2.2.1. Preparation of Solutions

The preparation of the solutions required for the experiments is based on the stock solutions of nitrate and phosphate anions. The stock solution of nitrate anions at 1.0000 g/L is obtained by dissolving 1.3492 g of sodium nitrate in 1 L of deionized water. The stock solution of phosphate anions at 1.0000 g/L is prepared by dissolving 1.4949 g of disodium phosphate in 1 L of deionized water.

From the stock solutions prepared, solutions of nitrate and phosphate anions of 10 mg/L, 30 mg/L, or 50 mg/L are obtained in deionized water.

The concentrations of nitrate and phosphate ions, considered in shallow water, starting from the hypothesis that they are close to the accepted limits for surface waters, were chosen in the range of 10–50 mg/L, approx. 10 mg/L for nitrate ions and approx. 50 mg/L for phosphate ions.

The solutions of nitrate and phosphate anions with *p*H 4, 7, and 11 are prepared using the appropriate amounts of anions and dissolving them in 10^−4^ mol/L hydrochloric acid solution for *p*H = 4, deionized water for *p*H = 7, or 10^−3^ mol/L sodium hydroxide solution for *p*H = 11. The *p*H is monitored with an electronic *p*H meter (Hanna Instruments Ltd., Leighton Buzzard, UK) equipped with a glass membrane electrode. *p*H corrections are made with solutions of concentration 10^−1^ mol/L hydrochloric acid or 10^−1^ mol/L sodium hydroxide. Under the proposed experimental conditions, a deviation of ±0.2 *p*H units was allowed, which covers the buffer effect given by phosphate anions.

#### 2.2.2. Obtaining Composite Membranes

The source of cellulose acetate containing silver nanoparticles (Agnp) was given by cinematographic films (exposed or unexposed to light). Cinematographic films were selected from eleven samples considered representative as raw material. To obtain a 2% cellulose acetate solution in dimethylformamide, 20 g of photographic film that was washed, dried, and ground to submicron sizes in a colloidal ball mill (Retsch 100) was dissolved in 980 g of dimethylformamide (DMF), with a gray solution (CA–Agnp–DMF) being obtained. Solutions of 4% and 6% cellulose acetate in DMF were also obtained in the same way.

To study the effect of Agnp concentration on nanofiltration, three types of film material were selected. One sample was chosen from light-exposed and image-forming films; the second sample was made from exposed and unexposed films; and the third sample was made from fully exposed (veiled) film. For atomic absorption spectroscopy (AAS) of silver in selected samples, 250 g of cinematographic film was mineralized with 250 g of nitric acid, after which the obtained solution was transferred to 500 mL of deionized water. The calibration curve for the chosen analysis method was made based on a stock solution of 1.0000 g/L silver ions (1.5747 g AgNO_3_ dissolved in one liter of deionized water) by successive dilutions to 0.01 ppm; 0.10 ppm; 1.00 ppm; 10.00 ppm; and 100.00 ppm. The silver content of the prepared samples was determined by atomic absorption spectroscopy, obtaining concentrations of 7.3 ppm (σ = 0.198, for 11 determinations), 11.5 ppm (σ = 0.193, for 11 determinations), and 16.1 ppm (σ = 0.191, for 11 determinations). These concentrations are expressed in gravimetric ppm relative to the cellulose acetate in the casting solution.

**Figure 2:**
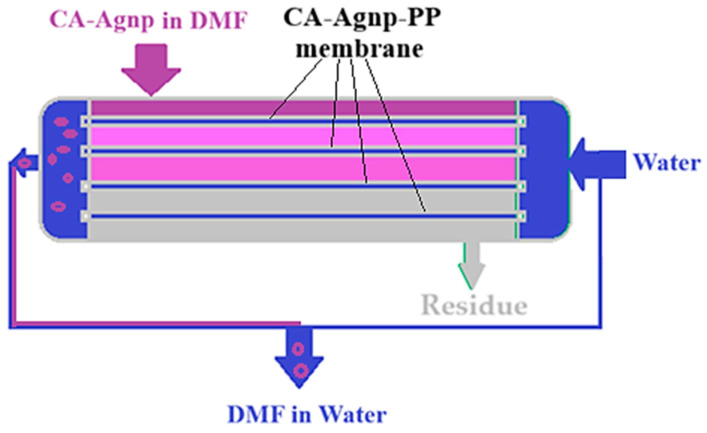
Obtaining CA–Agnp–PP composite membranes on the nanofiltration module (reproduced from [42], 2024, *U.P.B Sci. Bull.*).

To create CA–Agnp–PP composite membranes, the following steps were performed: phase inversion of the dispersed system (CA–Agnp–DMF) on and in the polypropylene (PP) hollow fibers of a nanofiltration module and contact with deionized water circulating through the fibers (Figure 2).

The CA–Agnp–PP composite membrane is obtained, which is thus prepared for nanofiltration of solutions containing sodium nitrate and sodium phosphate.

#### 2.2.3. Nanofiltration of Phosphate and Sodium Nitrate Solutions

The aqueous solution subjected to nanofiltration was synthetically obtained by dissolving sodium phosphate and/or sodium nitrate in deionized water. The concentration of each species was 10 mg/L, 30 mg/L, or 50 mg/L. The nanofiltration solutions were obtained based on sodium salts to avoid a complexity of cations in the system.

**Figure 3:**
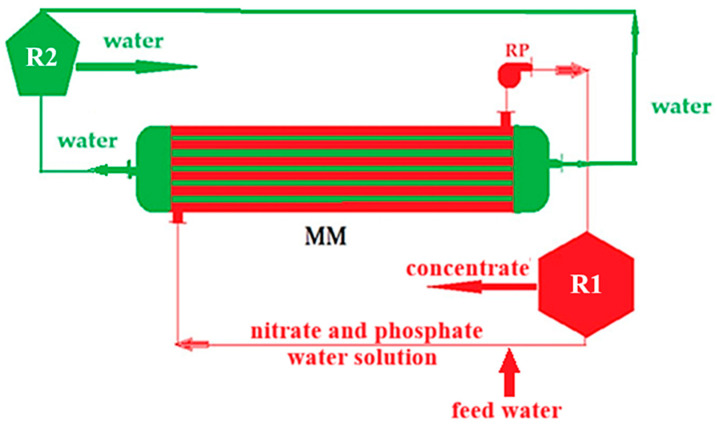
Nanofiltration of aqueous solution containing nitrate and phosphate ions: MM, membrane module; RP, recirculation pump; R1, raw water tank; and R2, nanofiltration water tank (permeate) (reproduced from [43], 2024, *Materials*).

Nanofiltration was performed by the module presented in Figure 3, in which the membranes were previously washed with deionized water. Complete washing was ascertained by checking the removal of bromide ion (remaining from unexposed cine films) from the permeate with silver nitrate solution.

The result of the nanofiltration was presented in the form of the permeate flux (*J*) (Equation (1)) and the retention, or the nanofiltration efficiency (*R*) (Equation (2)) [21,43]:

(1) $$J=\frac{V}{S\cdot \Delta t}(L/m^{2}\cdot h),$$

where *J* is permeate flux; *V* is permeate volume; *S* is membrane surface; and *Δt* is operating interval.

(2) $$R\%=\frac{\left(c_{0}-c_{f}\right)}{c_{0}}\cdot 100$$

where *R* is retention; *c*_0_ is concentration of feed solution; and *c_f_* is final concentration.

Analysis of nitrate and nitrate anions was performed with rapid tests: LCK349 Phosphorus total/Phosphate ortho and LCK339 Nitrate (Hach Lange GmbH, Düsseldorf, Germany).

### 2.3. Equipment

The microscopy studies, SEM and HR-SEM, were performed on a Hitachi S4500 system (Hitachi High—Technologies Europe GmbH, Mannheim, Germany) [21].

For the comparative study of the scanning electron microscopy (SEM) and energy-dispersive spectrum for the characteristic X–ray (EDAX) analyses, the membrane samples, subjected to the analysis, were visualized with the help of the FESEM–FIB workstation (scanning electron microscope with field emission electron and focused beam of ions), model Auriga (Carl Zeiss SMT, Oberkochen, Germany) [44].

The *p*H and anion concentrations were determined using a conductance cell or combined selective electrode (HI 4107, Hanna Instruments Ltd., Leighton Buzzard, UK) and a multi-parameter system (HI 5522, Hanna Instruments Ltd., Leighton Buzzard, UK) [45].

The determination of the concentration of nitrate or phosphate ions was performed using a CamSpec M550 Spectrophotometer (Spectronic CamSpec Ltd., Leeds, UK) [46].

An atomic absorption spectrometer AAnalyst 400 AA spectrometer (PerkinElmer Inc., Shelton, CT, USA) with WinLab32—AA software (PerkinElmer) and a single-element hollow-cathode lamp was used for silver concentration. The experimental parameters were 328.1 nm wavelength and 0.7 nm spectral bandwidth, at an operating current of 5 mA [47].

## 3. Results and Discussion

**Figure 4:**
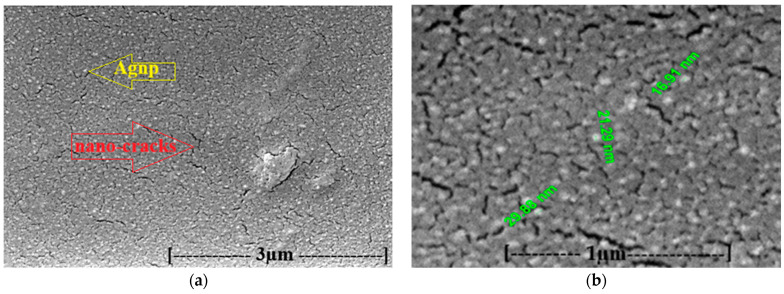
Scanning electron microscopy of the used cinematographic film: (**a**) yellow arrow—silver nanoparticles (Agnp); red arrow—nano-cracks of the cellulose acetate substrate; (**b**) dimensions of silver nanoparticles (depicted in green).

The black-and-white cinematographic films are now history and have become waste for two reasons: physical–chemical wear of the cellulose acetate substrate during archiving (Figure 4a) and translating information from motion pictures onto electronic media.

This waste is difficult to biodegrade due to the content of silver nanoparticles [21] whose size is 23 ± 7 nm (Figure 4b). It becomes necessary for physically and/or morally worn black-and-white cinematographic films to be recycled. A bold idea would be to recover the silver and then reuse the cellulose acetate. This idea is not feasible because silver is in too small a quantity to be technically and economically viable.

Starting from the results achieved in our team regarding the recycling of cinematographic films as membranes for the pertraction of hydrogen sulfide and organic sulfur compounds [21], this time, our goal was to obtain composite nanofiltration membranes based on cellulose acetate and silver, deposited on capillary membranes (hollow fiber) made of polypropylene as a support, and to test them for the removal of phosphate and nitrate ions found simultaneously in dilute aqueous solution.

The work on nanofiltration of solutions containing phosphate and nitrate anions through CA–Agnp–PP composite membranes was carried out as follows: obtaining and morphological and compositional characterization of membranes; determination of process characteristics using pure water; and determination of retention and fluxes in nanofiltration of phosphate and nitrate solutions.

### 3.1. Morphological and Compositional Characterization of the Obtained Membranes

**Figure 5:**
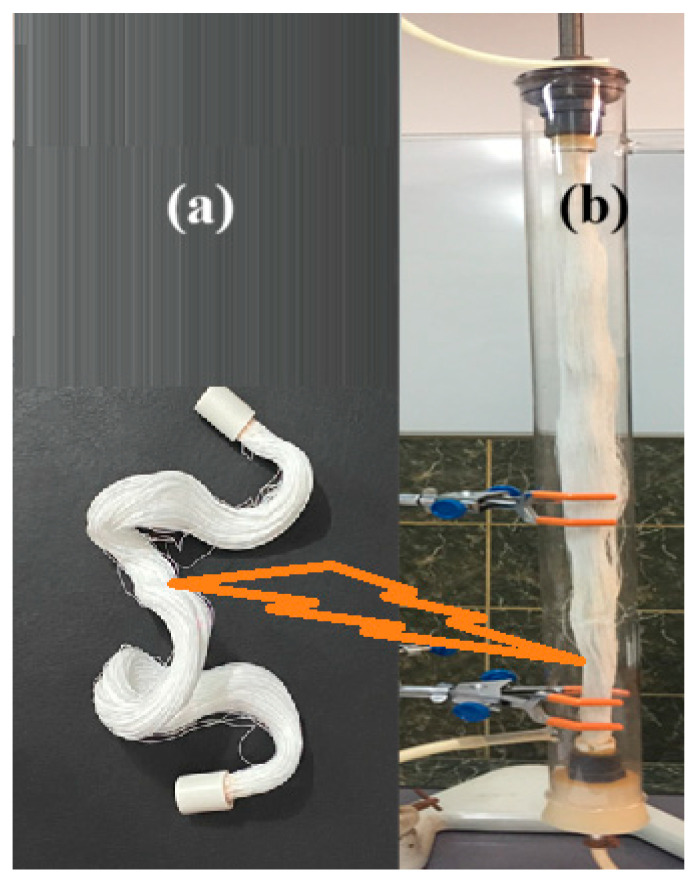
Hollow fiber ultrafiltration membrane: (**a**) the fiber bundle and (**b**) assembly into a module.

To obtain the composite membranes, a polypropylene support in the form of a hollow fiber (Figure 5a) was used, which has a porosity specific to membrane contactors and can be easily assembled into a module (Figure 5b) that we chose so as to ensure a filtering surface of 1 m^2^.

**Figure 6:**
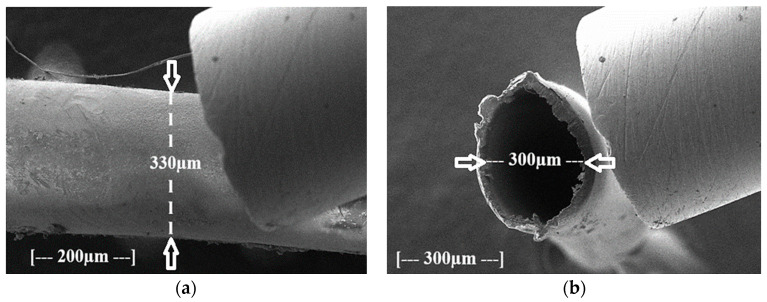
Scanning electron microscopy (SEM) of a hollow fiber membrane: (**a**) view; (**b**) section.

**Figure 7:**
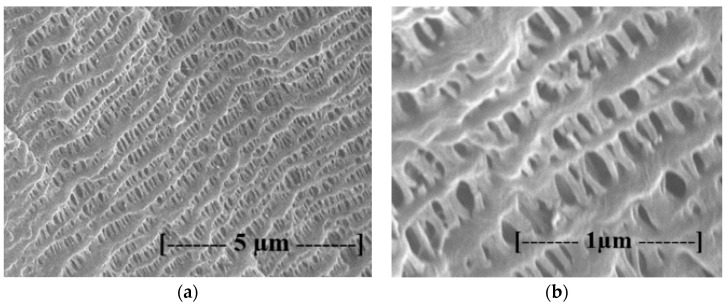
Scanning electron microscopy for the section of the polypropylene support membrane: (**a**) section and (**b**) section detail.

The morphological appearance of polypropylene support membranes shows an outer fiber diameter of about 330 µm (Figure 6a) and an inner diameter of approximately 300 µm (Figure 6b). The specific porosity of the ultrafiltration (or membrane contactors) of the polypropylene hollow fiber membrane is presented in Figure 7a,b.

The porosity of the hollow fiber polypropylene membranes indicates that they can be used for pertraction or in membrane contactors [47,48,49,50] (Figure 7a,b).

**Figure 8:**
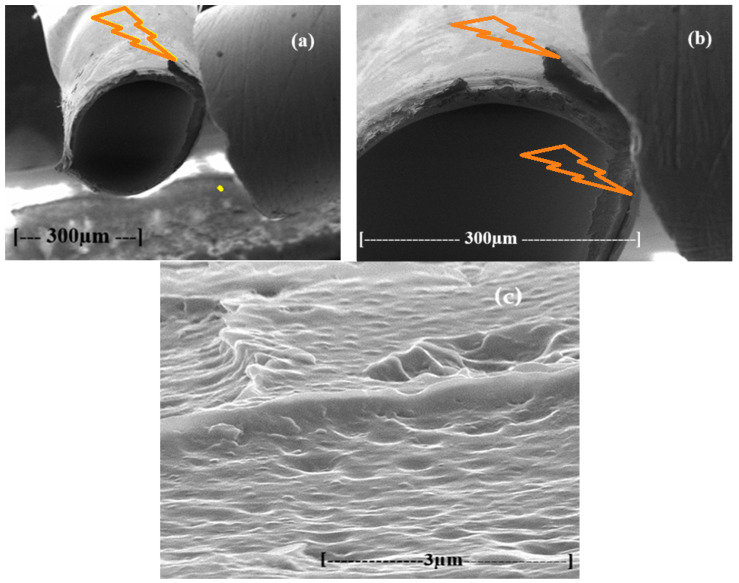
Scanning electron microscopy for the CA–Agnp–PP composite membrane: (**a**) section; (**b**) section detail, where arrows indicate the selective surface layer; (**c**) detail of the composite membrane surface (magnification of ×50,000).

Contacting the dispersion formed by cellulose acetate and silver nanoparticles through the support membranes with deionized water led to phase inversion, obtaining membranes with the morphology presented in Figure 8a, in which the deposition of the nanofiltration layer is observed (Figure 8b). The orange arrows highlight the deposited layer of cellulose acetate and silver nanoparticles. Figure 8c presents the surface of the composite membrane (at a magnification of ×50,000), in which no pores can be observed but in which silver nanoparticles covered by cellulose acetate appear.

**Figure 9:**
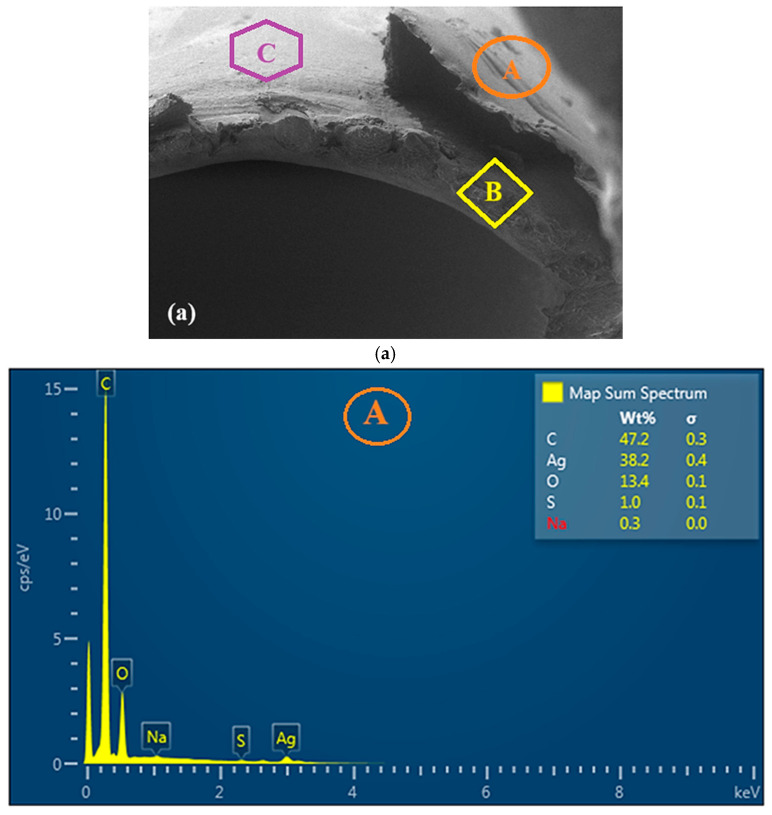

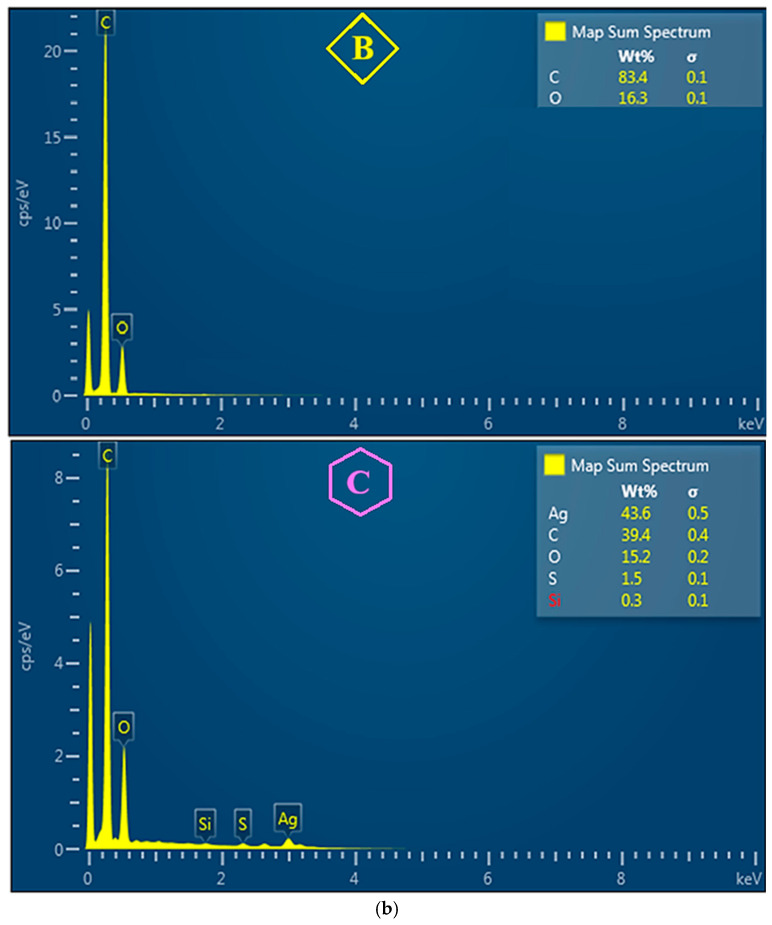
Sectional detail of CA–Agnp–PP composite membrane: (**a**) membrane with EDAX characterized areas; (**b**) EDAX for the examined surfaces (A, B, and C).

The EDX analysis of the membranes aimed at the composition of the selective (surface) layer and the polypropylene substrate. For the characterization of the membrane, the section in which the superficial nanofiltration layer is also highlighted was chosen (Figure 9a). The cross-sectional image shows the areas that were subjected to EDX analysis as follows: (bA) is the surface of the superficial layer; (bB) is the surface of the propylene substrate section; and (bC) is the surface of the superficial layer (the exfoliated part).

The analyses performed reveal that the surface of the superficial layer and the surface of the section of the superficial layer (the exfoliated part) contain (see Table 1) silver, carbon, oxygen, and sulfur, but also impurities, at trace levels, of sodium and silicon.

The surface (A) of the superficial layer is composed predominantly of silver, carbon, and oxygen, which are the expected components of cellulose acetate coming from cinematographic films. The presence of sulfur is most likely related to bisulfite developing solutions, which lead to residual sulfur (most likely silver sulfur). The presence of sodium is not unusual because it is a universal contaminant whose traces can be quite difficult to remove.

The section of the polypropylene support (B) indicates the presence of cellulose acetate in the pores of the support membrane, because along with carbon, we also have an appreciable concentration of oxygen. It is worth noting that nanometric silver does not penetrate the pores of the polypropylene hollow fiber membrane, which is demonstrated by its absence in the spectrum, but also by the lack of sulfur, which otherwise accompanies it in the other two analyses.

The section of the superficial layer (exfoliated part) (C) has a qualitative composition similar to that of the surface of this layer but slightly modified quantitatively. The absence of sodium, however, should be noted (most likely being eluted during the preparation of the membranes). The presence of silicon is accidental, most likely coming from the membrane cutting device during the preparation of the samples for scanning electron microscopy.

Following the EDX analysis of the cellulose acetate–silver nanoparticle–polypropylene (CA–Agnp–PP) composite membrane, the following can be stated:

• The composite membrane has a superficial layer composed of cellulose acetate and silver nanoparticles;
• The high concentration of silver shows that it is retained and distributed on the membrane surface during preparation;
• The superficial concentration of silver is very high compared to that in the solution obtained from cinematographic films, about 9 ppm compared to cellulose acetate;
• The membrane support (polypropylene) also contains cellulose acetate in its pores, highlighted by the presence of oxygen;
• Silver nanoparticles do not penetrate the pores of the polypropylene support;
• The presence of residual sulfur from the cinematographic film treating process was found;
• The presence of trace impurities (sodium and silicon) is related to sampling of the cinematographic films.

### 3.2. Determination of Nanofiltration Characteristics Through the CA–Agnp–PP Membrane Using Deionized Water

**Figure 10:**
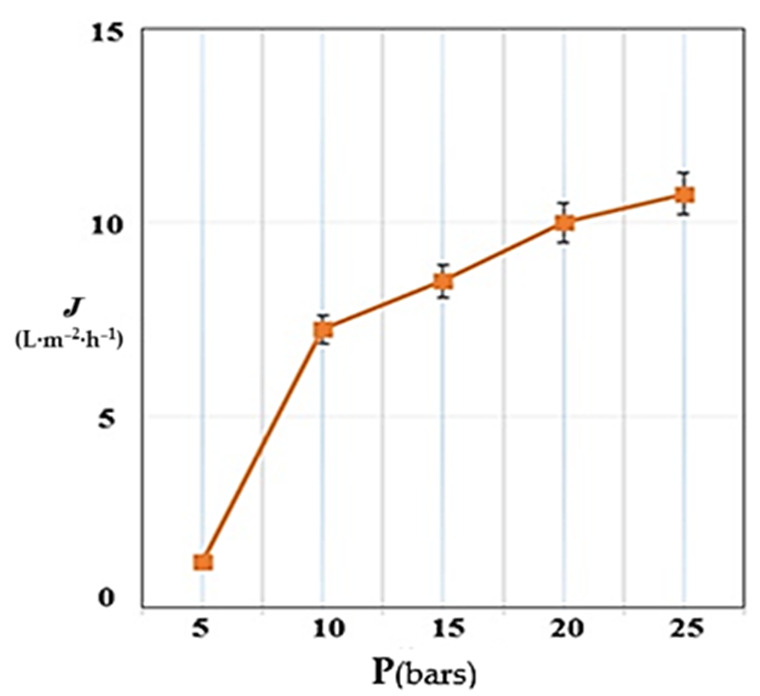
Dependence of deionized water flux on operating pressure.

The determination of permeate flows as a function of the working pressure (Figure 10) was carried out at a flow rate of 120 L/h of deionized water achieved with the recirculation pump (RP) (Figure 3). The working pressure is achieved using an air cylinder connected to the R1 tank, thus completing the supply water circuit. The nano-filtered water flow (permeate) was determined in the range of 5 to 25 bars. In Figure 10, we can see a significant increase in the flux in the range of 5–10 bars, after which the slope of the curve decreases. Increasing the pressure above 15 bars is not desirable because it reduces the technical and economic interest. Implementing a process at high pressure raises technical problems that are not compensated by the increase in permeate flux. The data presented in Figure 10 allow for the evaluation of an average permeability of 0.6 L·m^−2^·h^−1^·bar^−1^.

**Figure 11:**
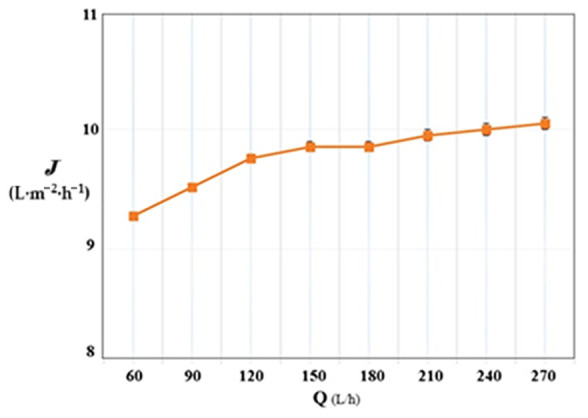
Dependence of deionized water flux on the flow rate through the fibers of the nanofiltration module.

The determination of permeate fluxes as a function of the flow regime was performed at a pressure of 15 bars (Figure 11). The flow regime was varied by changing the flow rate (Q) of the feed water through the module fibers. The increase in permeate flux occurs differently in the range of 60–120 L/h when the slope is high, compared to the range 120–270 L/h when the slope of the curve is significantly reduced.

It can be said that in the first part of the flow interval, the flow changes from laminar (Re < 2000) to a turbulent regime (Re > 4000) (Equation (3)):

(3) $$Re=\frac{v\cdot d}{\nu }$$

where *Re* is the Reynolds number; *v* is the water flow velocity through the nanofiltration module; *ν* is the kinematic viscosity of water (1.01 × 10^−6^ m^2^/s); and *d* is the equivalent diameter of the flow section (0.92 m).

In the second part of the curve, the flow increase is much reduced because the turbulent regime is maintained within the same parameters (Re >> 4000).

### 3.3. Determination of Permeate Flow and the Retention for CA–Agnp–PP Composite Membranes

To determine the performance of the prepared membranes, two working directions were approached: varying the characteristics of the casting solution from which the membranes were prepared and changing the feed solution parameters.

The constant parameters in the study were the working pressure, 15 bars, and the feed solution flow rate, 120 L/h.

Table 2 summarizes the results regarding permeate flux, nitrate anion, and phosphate anion retention depending on the concentration of cellulose acetate (C_CA_) and, respectively, silver particles (C_Agnp_), in the casting solution.

The variation of cellulose acetate concentration to 2%, 4%, and 6% (Table 2) led to a decrease in the flux value. Thus, from 15.2 L·m^−2^·h^−1^ for the 2% CA membrane and 10.7 L·m^−2^·h^−1^ for the 4% CA membrane, it reached 4.4 L·m^−2^·h^−1^ for the 6% CA membrane. The significant decrease in the flux value with increasing polymer concentration in the casting solution can be explained by the increase in the density of the surface layer of the formed membrane, but also by its thickness [46].

The retention of nitrate and phosphate anions increases due to the increase in the concentration of cellulose acetate in the casting solution. For the nitrate anion, the retention increases from 63.7%, obtained with the 2% CA membrane, to 96.5% with the 6% CA membrane. At the same time, the retention of phosphate anions increases from 75.2% for the 2% CA membrane to 99.0% for the 6% CA membrane.

Choosing the concentration of cellulose acetate in the casting solution will be a process of evaluating the need to obtain a high flux and a retention suitable to the needs. From the data obtained by us, the concentration of 4% CA in the casting solution is the one that achieves the flux–retention trade-off.

Increasing the concentration of silver nanoparticles (Agnp) in the casting solution (Table 2) causes a slight decrease in the flux from 11.3 L·m^−2^·h^−1^, for the membrane with 7.3 ppm Agnp in casting solution, to 9.7 L·m^−2^·h^−1^ for the 16.1 ppm Agnp membrane. For the retention values, an increase of approximately 3% was observed for both the nitrate and the phosphate anions, simultaneously with the increase in the Agnp concentration in the casting solution.

For the Agnp concentration in the casting solution, an increase would be desirable to ensure a higher retention of anions at relatively constant permeate flows. This parameter is determined by the quality of the raw material, which is not controllable from this point of view.

The modification of the feed solution parameters consisted of varying the *p*H and the anion concentration (Table 3).

The permeate flux decreases slightly both with the increase in *p*H and with the increase in the anion concentration in the feed solution, being approximately 11 L·m^−2^·h^−1^. The value obtained is specific to the nanofiltration of dilute aqueous solutions and depends on the hydration of the membrane, but also on the anions considered [51,52].

The variation in retention as a function of feed solution *p*H is much more relevant. Thus, for both the nitrate anion and the phosphate anion at a concentration of 30 mg/L, the increase in retention is remarkable, being about 5%, with the increase in *p*H from 4 to 7. A further increase in *p*H does not cause a significant increase in the retention of the two anions, being within the experimental error limits of approx. 95% for nitrate and approx. 99% for phosphate.

Increasing the anion concentration in the feed solution from 10 mg/L to 50 mg/L, at *p*H = 7, leads to a decrease in retention of approximately 3% for both anions.

The results presented are consistent with the literature data regarding the importance of ion hydration in the nanofiltration process [52,53,54], but also the hydration of cellulose derivatives [55].

The data obtained can also be explained by the behavior of both sodium nitrate (Equation (4)) and disodium phosphate (Equation (5)) in the feed solution:

(4) NaNO_3_(s) + H_2_O ⇌ Na^+^(aq) + NO_3_^−^(aq)

(5) Na_2_HPO_4_(s) + H_2_O ⇌ 2Na^+^(aq) + HPO_4_^2−^(aq)

where s indicates the solid state and aq is the aqueous solution.

The equilibria in Equations (4) and (5) are completely shifted to the right because the two salts are completely soluble.

Although disodium phosphate (Na_2_HPO_4_) salt was used as a source of phosphate ions, when dissolved in water, all species derived from phosphoric acid (H_2_PO_4_^−^, HPO_4_^2−^ and PO_4_^3−^) can coexist, depending on the *p*H of the solution (Equations (6)–(8)):

(6) H_3_PO_4_^−^ + H_2_O ⇌ H_2_PO_4_^−^ + H_3_O^+^   *p*Ka_1_ = 2.15

(7) H_2_PO_4_^−^ + H_2_O ⇌ HPO_4_^2−^ + H_3_O^+^   *p*Ka_2_ = 7.20

(8) HPO_4_^2−^ + H_2_O ⇌ PO_4_^3−^ + H_3_O^+^   *p*Ka_3_ = 12.32

The chemical species present in the solution have different interactions with the CA–Agnp–PP composite membrane. Having a negative charge, the nitrate ion exhibits ion-dipole interactions with hydroxyl (-OH), ether (-O-), and carboxylate (-COOCH_3_) groups in cellulose acetate (Figure 1b). The anionic species (HPO_4_^2−^ and PO_4_^3−^) derived from phosphoric acid have ion–dipole interactions with hydroxyl (-OH), ether (-O-), and carboxylate (-COOCH_3_) groups, stronger than those of the nitrate ion due to their higher charges. Anions that contain protons (H_2_PO_4_^−^, HPO_4_^2−^) can also interact through the hydrogen atoms with hydroxyl (-OH), ether (-O-), and carboxylate (-COOCH_3_) groups. However, the interactions presented do not reach the strength of hydrogen bonds.

The interactions of anions with the cellulose acetate membrane containing silver nanoparticles are stronger than those of sodium cations in solution. Therefore, phosphate and nitrate anions will be rejected by the membrane, while sodium cations will pass through it, with the Donnan effect, zeta potential, and negative charge effect being favorable to anionic nanofiltration [56].

The molar mass of phosphate anions is much higher than that of nitrate anions, and on the other hand, the crystallization water molecules only appear for salts derived from phosphate anions. These parameters lead to an increase in the size of the ionic aggregates of phosphates in solution, which is beneficial to their separation by nanofiltration (Table 4).

The presented aspects justify the higher retention (***R***) of phosphate anions compared to nitrate ions (Table 3). The presence of silver nanoparticles, which have positive charges on the outside, also favors the retention of phosphate anions, which have higher charges. At a *p*H below 12 (Table 3), protonated anions exhibit ion–ion interactions with the silver nanoparticles.

Ion–ion interactions and the formation of silver phosphate led to an increased retention, with increasing concentration of silver nanoparticles in the membrane (Table 2).

As a consequence of the presented aspects, it results that the operation must be performed at a high *p*H of the feed solution and a high concentration of silver nanoparticles in the membrane (Table 2 and Table 3).

To be recommended for applications, membranes must have a stable behavior during processing. To determine the variation of flux and retention as a function of time, the following parameters were established: working pressure, 15 bars; a feed solution flow rate of 120 L/h; a concentration of nitrate and phosphate anions equal to 30 mg/L; a *p*H of the feed solution of 7.0 ± 0.2; a membrane obtained from a casting solution of 4% cellulose acetate and 11.5 ± 0.2 ppm Agnp; experiments performed daily, while keeping the same membrane; and a feed solution that is refreshed daily (600 L/day).

**Figure 12:**
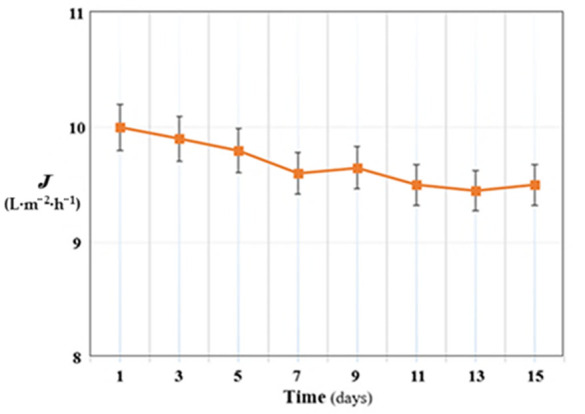
Time dependence of nanofiltration flux of aqueous nitrate and phosphate solution.

From Figure 12, a permanent but slow decrease in the flux can be highlighted from approx. 10.2 L·m^−2^·h^−1^ on the first day to approx. 9.5 L·m^−2^·h^−1^ on the fifteenth day of operation. The decrease in flux is probably determined by membrane fouling because the phosphate anions (PO_4_^3−^) form a precipitate layer (Ag_3_PO_4_) with the silver cations on the surface of the nanoparticles, with the solubility product (Ks) shown in Equation (9):

(9) 3Ag^+^ +PO_4_^3−^ ⇌ Ag_3_PO_4_   Ks = 1.8 × 10^−18^

**Figure 13:**
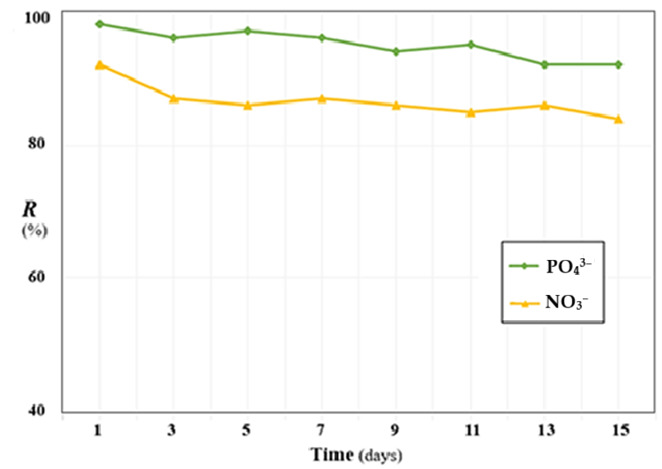
Dependence of the retention of phosphate and nitrate anions from a 30 ppm concentration solution on the operating time.

The determination of retention during nanofiltration of phosphate and nitrate solutions (Figure 13) was carried out under the experimental conditions established for determining the flux variation as a function of time.

Over the entire time interval, the retention is significantly higher for the phosphate anion than for the nitrate anion. The retention of both anions decreases from the first day, when it is about 98.1% for the phosphate anion and about 93.2% for the nitrate anion, to 95.0% for the phosphate anion and 84.3% for the nitrate anion on the 15th day of experiments. The error in determining retention is ±0.2% for the nitrate anion and ±0.3% for the phosphate anion and is given by both the testing of samples taken and the analytical errors of determination.

The decrease in retention during operation can be explained similarly to that from the decrease in flux, namely, the formation of silver phosphate on the surface of the nanoparticles. On the other hand, the formation of silver phosphate prevents the possible loss of membrane material (toxic silver ions) in the permeate. The presence of silver in drinking water is regulated by the World Health Organization (WHO) at 0.1 mg/L.

### 3.4. Application and Research Perspectives of Nanofiltration with CA–Agnp–PP Membranes

Using nanofiltration for obtaining drinking water is recommended in isolated areas such as farms and households in locations where it is common to extract water from shallow depths. This water source may be contaminated from local sources (animals, birds, fertilizers), even with nitrate and phosphate anions. Presumptive concentrations would be 30 ppm from each anion in the feed water from the shallow well.

Drinking water needs for a family of five can reach about 50 L/day, including water for drinking, cooking, and washing the face and mouth.

The results presented in the previous subsections show that membranes obtained from cinematographic films membranes (CA–Agnp–PP) and assembled into a module with a surface area of approximately 1 m^2^ can meet these needs, at an operating pressure of 15 bars and a feed water flow rate of 120 L/h, with the rejection being 98% for phosphate and 95% for nitrate, with a flux of 10 L·m^−2^·h^−1^. The data presented shows that during a 5 h operating interval, the drinking water needs of an entire family of five can be covered.

**Figure 14:**
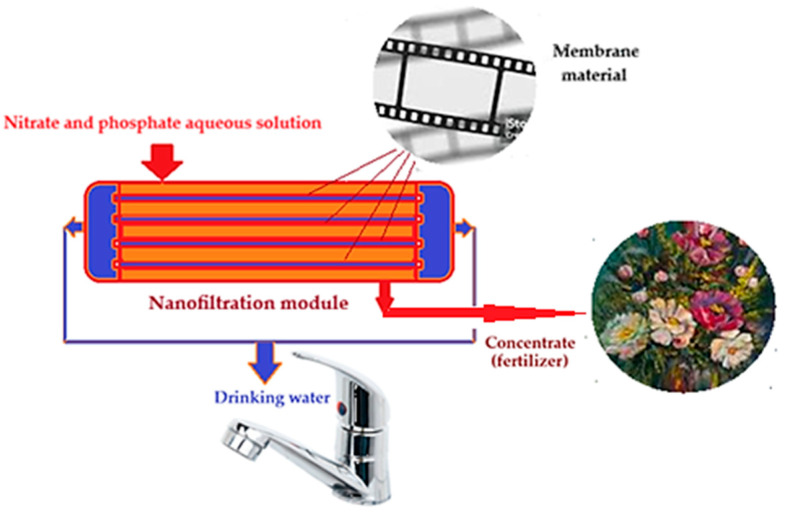
Schematic presentation of obtaining drinking water and fertilizer through nanofiltration.

The concentrate obtained from nanofiltration can be used as fertilizer for ornamental flowers or in the vegetable garden (Figure 14).

Of course, the application presented is only a suggestion, but it is important for locations such as houses in mountainous and hilly areas, agricultural or livestock farms in the plains, and river deltas or in cases of flooding.

The practical and applied aspects that this study did not consider, and which remain to be addressed in future research, are determination of several membrane characteristics, such as zeta potential, pore size distribution, surface roughness, contact angle, and porosity; production of large and representative batches of cinematographic material, thus ensuring the reproducibility of the raw material for membrane preparation; leaching of silver ions during nanofiltration; testing of real water sources when the influence of a complex matrix of anions and cations on nanofiltration can be studied; and fouling behavior, cleaning efficiency, or flux recovery [13,19,56,57].

Also, one of the important issues for future research is the replacement of dimethylformamide (toxic) with an environmentally friendly membrane solvent (green solvent) [13,58].

## 4. Conclusions

Finding sources of raw material for obtaining membranes also involves recycling various polymeric waste.

For one of the most important artificial membrane polymers, cellulose acetate, various wastes were used, including cigarette filters or cinematographic films.

This paper presents the production of nanofiltration membranes using black-and-white cinematographic films and their testing in the nanofiltration of waters contaminated with nitrate and phosphate anions, in order to obtain drinking water for isolated areas.

Composite nanofiltration membranes were obtained by phase inversion of the cellulose acetate (cinematographic film) solution in dimethylformamide with deionized water. The composite membranes made on polypropylene fiber support have cellulose acetate and silver nanoparticles from cinematographic film as a selective layer.

In this study, the operating parameters (working pressure, flow rate, and *p*H of feed water) were varied, as well as the parameters that determine membrane formation (cellulose acetate concentration and silver nanoparticle concentration).

The results show that under accessible working conditions (a pressure of 15 bars and a feed water flow rate of 120 L/h, *p*H = 7, rejection of 98% for phosphate and 95% for nitrate, and a flux of 10 L·m^−2^·h^−1^), the drinking water needs of a family of five (50 L/day) can be covered in an operating interval of 5 h.

The presented study may constitute the premise for obtaining drinking water from shallow waters in isolated areas. The presence of silver nanoparticles in membranes may be a solution for eliminating biofouling, which would lead to a decrease in transmembrane flux.

## Figures and Tables

**Table 1:** EDX analysis on the surface and in the section of composite membranes.

Atom		Silver (%)	Carbon (%)	Oxygen (%)	Sulfur (%)	Sodium (%)	Silicon (%)
Surface	A	38.2 ± 0.3	47.2 ± 0.4	13.4 ± 0.1	1.0 ± 0.1	0.3 ± 0.0	-
	B	-	83.4 ± 0.1	16.3 ± 0.1	-	-	-
	C	43.6 ± 0.5	39.4 ± 0.4	15.2 ± 0.2	1.5 ± 0.1	-	0.3 ± 0.1

**Table 2:** Dependence of flux and retention on the concentration of polymer (**C_CA_**) and silver nanoparticles (**C_Agnp_**) in the casting solution.

Parameter	C_CA_ (%)			C_Agnp_ (ppm)		
	2	4	6	7.3	11.5	16.1
Flux (L·m^−2^·h^−1^)	15.2 ± 0.4	10.7 ± 0.4	4.4 ± 0.4	11.3 ± 0.4	10.7 ± 0.4	9.7 ± 0.4
Retention nitrate (%)	63.7 ± 0.2	95.0 ± 0.2	96.5 ± 0.2	92.5 ± 0.2	95.0 ± 0.2	96.1 ± 0.2
Retention phosphate (%)	75.2 ± 0.3	98.1 ± 0.3	99.0 ± 0.3	95.2 ± 0.3	98.1 ± 0.3	99.2 ± 0.3

**Table 3:** Dependence of flux and retention on *p*H and ionic concentration of the feed solution (FS).

Parameter	*p*H			C_FS_ (mg/L) ^(1)^		
	4	7	11	10	30	50
Flux (L·m^−2^·h^−1^)	11.2 ± 0.4	10.7 ± 0.4	10.4 ± 0.4	11.3 ± 0.4	10.7 ± 0.4	9.9 ± 0.4
Retention nitrate (%)	90.7 ± 0.2	95.0 ± 0.2	95.8 ± 0.2	96.5 ± 0.2	95.0 ± 0.2	93.2 ± 0.2
Retention phosphate (%)	95.2 ± 0.3	98.1 ± 0.3	99.1 ± 0.3	99.2 ± 0.3	98.1 ± 0.3	96.2 ± 0.3

^(1)^ C_FS_ = concentration of the feed solution.

**Table 4:** Hydration molecules of nitrate and phosphate anions.

Salt	NaNO_3_	NaH_2_PO_4_	Na_2_HPO_4_	Na_3_PO_4_
H_2_O ^(1)^	–	2	12	10

^(1)^ Water molecules for salt crystallization.

## Data Availability

The original contributions presented in this study are included in the article. Further inquiries can be directed to the corresponding authors.

## References

[1] Wang Y., Wei G. (2024). Recent Trends in Polymer Membranes: Fabrication Technique, Characterization, Functionalization, and Applications in Environmental Science (Part I). Polymers.

[2] Satchanska G., Davidova S., Petrov P.D. (2024). Natural and Synthetic Polymers for Biomedical and Environmental Applications. Polymers.

[3] Oprea M., Voicu S.I. (2023). Cellulose Acetate-Based Materials for Water Treatment in the Context of Circular Economy. Water.

[4] Mulder M. (1996). Basic Principles of Membrane Technology.

[5] Oprea M., Voicu S.I. (2020). Recent Advances in Applications of Cellulose Derivatives-Based Composite Membranes with Hydroxyapatite. Materials.

[6] An Y., Li F., Di Y., Zhang X., Lu J., Wang L., Yan Z., Wang W., Liu M., Fei P. (2024). Hydrophobic Modification of Cellulose Acetate and Its Application in the Field of Water Treatment: A Review. Molecules.

[7] Morsi R.E., Corticelli F., Morandi V., Gentili D., Cavallini M., Figoli A., Russo F., Galiano F., Aluigi A., Ventura B. (2023). Influence of the Fabrication Conditions on the Physical Properties and Water Treatment Efficiency of Cellulose Acetate Porous Membranes. Water.

[8] Koriem O.A., Kamel A.M., Shaaban W., Elkady M.F. (2022). Enhancement of Dye Separation Performance of Eco-Friendly Cellulose Acetate-Based Membranes. Sustainability.

[9] Aldalbahi A., El-Naggar M., Khattab T., Abdelrahman M., Rahaman M., Alrehaili A., El-Newehy M. (2020). Development of Green and Sustainable Cellulose Acetate/Graphene Oxide Nanocomposite Films as Efficient Adsorbents for Wastewater Treatment. Polymers.

[10] Ounifi I., Guesmi Y., Ursino C., Santoro S., Mahfoudhi S., Figoli A., Ferjanie E., Hafiane A. (2021). Antifouling Membranes Based on Cellulose Acetate (CA) Blended with Poly(acrylic acid) for Heavy Metal Remediation. Appl. Sci..

[11] Asiri A.M., Petrosino F., Pugliese V., Khan S.B., Alamry K.A., Alfifi S.Y., Marwani H.M., Alotaibi M.M., Algieri C., Chakraborty S. (2022). Synthesis and Characterization of Blended Cellulose Acetate Membranes. Polymers.

[12] Kuzminova A., Dmitrenko M., Dubovenko R., Puzikova M., Mikulan A., Korovina A., Koroleva A., Selyutin A., Semenov K., Su R. (2024). Development and Study of Novel Ultrafiltration Membranes Based on Cellulose Acetate. Polymers.

[13] Islam M.D., Uddin F.J., Rashid T.U., Shahruzzaman M. (2023). Cellulose acetate-based membrane for wastewater treatment—A state-of-the-art review. Mater. Adv..

[14] Elbadawi N.A., Ramadan A.R., Esawi A.M.K. (2022). Studying the Effect of Shortening Carbon Nanotubes via Ball Milling on Cellulose Acetate Nanocomposite Membranes for Desalination Applications. Membranes.

[15] Abu-Zurayk R., Alnairat N., Khalaf A., Ibrahim A.A., Halaweh G. (2023). Cellulose Acetate Membranes: Fouling Types and Antifouling Strategies—A Brief Review. Processes.

[16] Li T., Wang Y., Wang X., Cheng C., Zhang K., Yang J., Han G., Wang Z., Wang X., Wang L. (2022). Desalination Characteristics of Cellulose Acetate FO Membrane Incorporated with ZIF-8 Nanoparticles. Membranes.

[17] Chaithra K.P., Varghese A., Vinod T.P., Sunajadevi K.R.P. (2024). Multifunctional electrospun membranes incorporated with metal oxide nanoparticles, cellulose acetate, and polyvinylpyrrolidone for wastewater treatment: Oil/water separation, dye adsorption, and dye degradation. Chem. Eng. J..

[18] Mahmodi G., Bafti R.R., Boroujeni N.I., Pradhan S., Dangwal S., Sengupta B., Vatanpour V., Sorci M., Fathizadeh M., Bikkina P. (2023). Improving cellulose acetate mixed matrix membranes by incorporating hydrophilic MIL-101 (Cr)-NH_2_ nanoparticles for treating dye/salt solution. Chem. Eng. J..

[19] Santoro S., Occhiuzzi J., Aquino M., Politano A., Straface S., D’Andrea G., Carrillo C., Mallada R., Garcia A., Estay H. (2024). Green photocatalytic mixed matrix membranes for simultaneous arsenic photo-oxidation and water recovery via membrane distillation. Sep. Purif. Technol..

[20] Rodrigues Filho G., Monteiro D.S., da Silva Meireles C., de Assunção R.M.N., Cerqueira D.A., Barud H.S., Ribeiro S.J., Messadeq Y. (2008). Synthesis and characterization of cellulose acetate produced from recycled newspaper. Carbohydr. Polym..

[21] Nechifor A.C., Cotorcea S., Bungău C., Albu P.C., Pașcu D., Oprea O., Grosu A.R., Pîrțac A., Nechifor G. (2021). Removing of the Sulfur Compounds by Impregnated Polypropylene Fibers with Silver Nanoparticles-Cellulose Derivatives for Air Odor Correction. Membranes.

[22] Torkashvand J., Saeedi-Jurkuyeh A., Rezaei Kalantary R., Gholami M., Esrafili A., Yousefi M., Farzadkia M. (2022). Preparation of a cellulose acetate membrane using cigarette butt recycling and investigation of its efficiency in removing heavy metals from aqueous solution. Sci. Rep..

[23] Friuli M., Grazioli C., Sattar N., Zia J., Del Sole R., Mergola L., Pal S., Licciulli A., Demitri C., Sannino A. (2025). Eco-friendly recovery of cellulose acetate from combusted cigarette filters and reuse for membrane fabrication. Waste Manag..

[24] Slejko E.A., Tuan A., Scuor N. (2024). From waste to value: Characterization of recycled cellulose acetate for sustainable waste management. Waste Manag. Bull..

[25] European Union (2015). Council Directive 2000/60/EC of the European Parliament and of the Council of 23 October 2000 Establishing a Framework for Community Action in the Field of Water Policy. https://eur-lex.europa.eu/eli/dir/2000/60/oj.

[26] European Union (2015). Council Directive 98/83/EC of 3 November 1998 on the Quality of Water Intended for Human Consumption. EUR-Lex-01998L0083–20151027-EN. https://data.europa.eu/eli/dir/1998/83/2015-10-27.

[27] Abdoli S., Asgari Lajayer B., Dehghanian Z., Bagheri N., Vafaei A.H., Chamani M., Rani S., Lin Z., Shu W., Price G.W. (2024). A Review of the Efficiency of Phosphorus Removal and Recovery from Wastewater by Physicochemical and Biological Processes: Challenges and Opportunities. Water.

[28] Huang L., Lu Z., Xie T., Wang L., Mo C. (2022). Nitrogen and phosphorus removal by coupling Anaerobic ammonia oxidation reaction with algal-bacterial symbiotic system. J. Environ. Chem. Eng..

[29] Derco J., Žgajnar Gotvajn A., Guľašová P., Kassai A., Šoltýsová N. (2024). Nutrient Removal and Recovery from Municipal Wastewater. Processes.

[30] Ma J., Wei W., Qin G., Xiao T., Tang W., Zhao S., Jiang L., Liu S. (2022). Electrochemical reduction of nitrate in a catalytic carbon membrane nano-reactor. Water Res..

[31] Sun F.Y., Wang X.M., Li X.Y. (2013). An innovative membrane bioreactor (MBR) system for simultaneous nitrogen and phosphorus removal. Process Biochem..

[32] Velusamy K., Periyasamy S., Kumar P.S., Vo D.V.N., Sindhu J., Sneka D., Subhashini B. (2021). Advanced techniques to remove phosphates and nitrates from waters: A review. Environ. Chem. Lett..

[33] Nie J., Huang H., Rao P., Chen H., Du X., Wang Z., Zhang W., Liang H. (2023). Composite functional particle enhanced gravity driven ceramic membrane bioreactor for simultaneous removal of nitrogen and phosphorus from groundwater. Chem. Eng. J..

[34] Lüdtke K., Peinemann K.V., Kasche V., Behling R.D. (1998). Nitrate removal of drinking water by means of catalytically active membranes. J. Membr. Sci..

[35] Gao Q., Wang C.Z., Liu S., Hanigan D., Liu S.T., Zhao H.Z. (2019). Ultrafiltration membrane microreactor (MMR) for simultaneous removal of nitrate and phosphate from water. Chem. Eng. J..

[36] Fang D., Huang L., Xiao H., Wu G., Zeng Z., Wang X., Yang G., Shen F., Deng S., Ji F. (2023). Layered double hydroxide membranes for advanced removal of phosphate from wastewater. Chem. Eng. J..

[37] Ren L., Xu J., Dai R., Wang Z. (2023). Electrochemical removal and recovery of phosphorus from wastewater using cathodic membrane filtration reactor. Chin. Chem. Lett..

[38] Popova A., Rattanakom R., Yu Z.Q., Li Z., Nakagawa K., Fujioka T. (2023). Evaluating the potential of nanofiltration membranes for removing ammonium, nitrate, and nitrite in drinking water sources. Water Res..

[39] Tepuš B., Simonič M., Petrinić I. (2009). Comparison between nitrate and pesticide removal from ground water using adsorbents and NF and RO membranes. J. Hazard. Mater..

[40] Mehenktaş C., Arar Ö. (2023). Application of membrane processes for nitrate (NO_3_^−^) removal. Curr. Chin. Sci..

[41] Ghimpusan M., Nechifor G., Din I.S., Nechifor A.C., Passeri P. (2016). Application of Hollow Fibre Membrane Bioreactor Instead of Granular Activated Carbon Filtration for Treatment of Wastewater from Car Dismantler Activity. Mater. Plast..

[42] Man G.T., Albu P.C., Popescu (Stegăruș) D.I., Niculescu V.-C., Marinescu V.E., Nechifor A.C. (2024). Thorium recovery from the Tungsten welding electrodes by electrolysis and nanofiltration. U.P.B. Sci. Bull. Ser. B.

[43] Albu P.C., Pîrțac A., Motelica L., Nechifor A.C., Man G.T., Grosu A.R., Tanczos S.-K., Grosu V.-A., Nechifor G. (2024). Reduction in Olfactory Discomfort in Inhabited Premises from Areas with Mofettas through Cellulosic Derivative–Polypropylene Hollow Fiber Composite Membranes. Materials.

[44] Nechifor A.C., Albu P.C., Motelica L., Man G.T., Grosu A.R., Tanczos S.-K., Grosu V.-A., Marinescu V.E., Nechifor G. (2024). Thorium Recovery with Crown Ether–Polymer Composite Membranes. Appl. Sci..

[45] Ferencz A., Grosu A.R., Al-Ani H.N.A., Nechifor A.C., Tanczos S.-K., Albu P.C., Crăciun M.E., Ioan M.-R., Grosu V.-A., Nechifor G. (2022). Operational Limits of the Bulk Hybrid Liquid Membranes Based on Dispersion Systems. Membranes.

[46] Razvan A., Man G.T., Dumitru F., Pandele M., Trusca R., Motelica L., Nechifor G. (2024). Nanocomposite membranes prepared from cellulose acetate or polysulfone with Ag0 nanoparticles and nitron reagent for nitrate ion removal. Desalin. Water Treat..

[47] Dimulescu I.A., Nechifor A.C., Bǎrdacǎ C., Oprea O., Paşcu D., Totu E.E., Albu P.C., Nechifor G., Bungău S.G. (2021). Accessible Silver-Iron Oxide Nanoparticles as a Nanomaterial for Supported Liquid Membranes. Nanomaterials.

[48] Ramezani R., Di Felice L., Gallucci F. (2022). A Review on Hollow Fiber Membrane Contactors for Carbon Capture: Recent Advances and Future Challenges. Processes.

[49] Magnone E., Shin M.C., Park J.H. (2025). Polymeric Membrane Contactors for CO_2_ Separation: A Systematic Literature Analysis of the Impact of Absorbent Temperature. Polymers.

[50] Nechifor G., Păncescu F.M., Grosu A.R., Albu P.C., Oprea O., Tanczos S.-K., Bungău C., Grosu V.-A., Pîrțac A., Nechifor A.C. (2021). Osmium Nanoparticles-Polypropylene Hollow Fiber Membranes Applied in Redox Processes. Nanomaterials.

[51] Van der Bruggen B., Kim J. (2012). Nanofiltration of Aqueous Solutions: Recent Developments and Progresses. Advanced Materials for Membrane Preparation.

[52] Lu C., Chen Z., Wu Y., Zhang Y., Wang F., Hu C., Qu J. (2024). Response of ionic hydration structure and selective transport behavior to aqueous solution chemistry during nanofiltration. Environ. Sci. Technol..

[53] Suhalim N.S., Kasim N., Mahmoudi E., Shamsudin I.J., Mohammad A.W., Mohamed Zuki F., Jamari N.L.-A. (2022). Rejection Mechanism of Ionic Solute Removal by Nanofiltration Membranes: An Overview. Nanomaterials.

[54] Giacobbo A., Pasqualotto I.F., Machado Filho R.C.d.C., Minhalma M., Bernardes A.M., Pinho M.N.d. (2023). Ultrafiltration and Nanofiltration for the Removal of Pharmaceutically Active Compounds from Water: The Effect of Operating Pressure on Electrostatic Solute—Membrane Interactions. Membranes.

[55] Rojewska M., Jakubowska E., Szelejewska K., Nowaczyk M., Froelich A., Prochaska K., Osmałek T. (2026). Cellulose-Based Polymer Blends for Oral Mucoadhesion: Impact of Hydration and Surface Interactions. Polymers.

[56] Figueiredo A.S., Sánchez-Loredo M.G., de Pinho M.N., Minhalma M. (2025). Surface-Charge Characterization of Nanocomposite Cellulose Acetate/Silver Membranes and BSA Permeation Performance. Membranes.

[57] Aquino M., Santoro S., Di Profio G., La Russa M.F., Limonti C., Straface S., D’Andrea G., Curcio E., Siciliano A. (2023). Membrane distillation for separation and recovery of valuable compounds from anaerobic digestates. Sep. Purif. Technol..

[58] Di Luca G., Galiano F., Russo F., Tornaghi S., Di Nicolò E., Mancuso R., Gabriele B., Figoli A. (2025). Sustainable membrane preparation: Approaches using cellulose acetate as a biopolymer and ethyl lactate as a green solvent. ACS Sustain. Chem. Eng..

